# Identification of Whirly transcription factors in Triticeae species and functional analysis of *TaWHY1-7D* in response to osmotic stress

**DOI:** 10.3389/fpls.2023.1297228

**Published:** 2023-12-05

**Authors:** Hao Liu, Xiaoyu Wang, Wenbo Yang, Wenyan Liu, Yanfang Wang, Qin Wang, Yanhong Zhao

**Affiliations:** ^1^ College of Agriculture, Ludong University, Yantai, China; ^2^ College of Chemical and Biological Engineering, Shandong University of Science and Technology, Qingdao, China; ^3^ Institute of Cereal Crops, Henan Academy of Agricultural Sciences, Zhengzhou, China; ^4^ College of Life Science, Ludong University, Yantai, China; ^5^ Department of Biochemistry and Molecular Biology, Binzhou Medical University, Yantai, China

**Keywords:** Triticeae species, wheat, *Whirly* gene, gene expression, osmotic stress

## Abstract

Osmotic stress poses a threat to the production and quality of crops. *Whirly* transcription factors have been investigated to enhance stress tolerance. In this study, a total of 18 *Whirly* genes were identified from six Triticeae species, which were classified into *Whirly1* and *Whirly2*. The exon–intron structure, conserved motif, chromosomal location, collinearity, and regulatory network of *Whirly* genes were also analyzed. Real-time PCR results indicated that *TaWHY1* genes exhibited higher expression levels in leaf sheaths and leaves during the seedling stage, while *TaWHY2* genes were predominantly expressed in roots. Under PEG stress, the expression levels of *TaWHY1-7A*, *TaWHY2-6A*, *TaWHY2-6B*, and *TaWHY2-6D* were increased, *TaWHY1-7D* was reduced, and *TaWHY1-4A* had no significant change. All *TaWHY* genes were significantly up-regulated in response to NaCl stress treatment. In addition, TaWHY1-7A and TaWHY1-7D mainly enhanced the tolerance to oxidative stress in yeast cells. TaWHY2s mainly improved NaCl stress tolerance and were sensitive to oxidative stress in yeast cells. All TaWHYs slightly improved the yeast tolerance to d-sorbitol stress. The heterologous expression of *TaWHY1-7D* greatly improved drought and salt tolerance in transgenic *Arabidopsis*. In conclusion, these results provide the foundation for further functional study of *Whirly* genes aimed at improving osmotic stress tolerance in wheat.

## Introduction

Wheat (*Triticum aestivum* L.) is one of the most important staple crops worldwide and a major source of calories for the expanding world population. As a sessile organism, wheat has to suffer from a variety of adverse conditions during the growth and development stages, such as drought and salinization, which contribute to a great reduction in the overall wheat yield and quality (Gupta et al., 2020). Therefore, mining stress-resistant genes and developing improved varieties are the most important strategies to improve wheat yield and quality.

Whirly (WHY) proteins are plant-specific transcription factors binding to single-stranded DNA (ssDNA) to modulate growth and defense responses and located in the chloroplasts, mitochondria, and nucleus (Desveaux et al., 2005; Krupinska et al., 2022; Taylor et al., 2022). Whirly domain consists of four structural topologies, which are characterized by two antiparallel four-stranded *β*-sheets stabilized by a C-terminal helix-loop-helix motif (Desveaux et al., 2005; Cappadocia et al., 2013; Taylor et al., 2022). Due to the structural similarity with “whirligig,” Whirly transcription factors are named Whirly (Desveaux et al., 2005). The conserved “KGKAAL” motif in the Whirly domains exists extensively in higher plants, which participate in binding to ssDNA and hexamerization of the tetramers forming hollow sphere structures of 12 nm in diameter (Desveaux et al., 2002; Cappadocia et al., 2012). Additionally, Whirly proteins contain a conserved cysteine residue, which might play a vital role in the formation of disulfide bridges between two Whirly proteins (Foyer et al., 2014).

Whirly was initially identified as p24/PBF-2 protein that binds to the elicitor response element (ERE) on the promoter of the pathogen response gene *PR-10a* in potato (Desveaux et al., 2000). In *Arabidopsis*, AtWHY1 is targeted to chloroplasts and nucleus (Krause et al., 2005; Ren et al., 2017), which plays a crucial role in regulating telomere length homeostasis (Yoo et al., 2007), maintaining the stability of plastid genome (Marechal et al., 2009), modulating reactive oxygen species (ROS) homeostasis, controlling leaf senescence (Lin et al., 2019), and responding to salicylic acid (SA)-dependent disease resistance (Desveaux et al., 2004). AtWHY1 protein represses the expression of *WRKY53* and delays leaf senescence in *Arabidopsis* (Miao et al., 2013). AtWHY2 is located in the mitochondria and nucleus (Krause et al., 2005; Golin et al., 2020). Overexpression of *AtWHY2* leads to mitochondrial dysfunction, early accumulation of senescence-related transcripts (Marechal et al., 2008; Golin et al., 2020), slower growth of pollen tubes, elevation of mtDNA content, and ROS levels in pollen (Cai et al., 2015). AtWHY3 is targeted to chloroplasts, mitochondria, and nucleus in compensation for the lack or mutation of AtWHY1 and AtWHY2 (Krause et al., 2005; Golin et al., 2020). In tomato (*Solanum lycopersicum*), *SlWHY1* and *SlWHY2* can be induced by drought and salt stresses (Akbudak and Filiz, 2019). Overexpression of *SlWHY1* enhances heat and cold stress tolerance and reduces ROS levels in tomato (Zhuang et al., 2020a; Zhuang et al., 2020b), and SlWHY2 can maintain mitochondrial function under drought stress through interacting with SlRECA2 in tomato (Meng et al., 2020). MeWHY1/2/3 can interact with MeCIPK23 to activate abscisic acid (ABA) biosynthesis and regulate drought resistance in cassava (*Manihot esculenta*) (Yan et al., 2020). In barley (*Hordeum vulgare* L.), overexpression of HvWHY1 delays drought-induced leaf senescence (Manh et al., 2023).

*Whirly* genes have been identified in various plant species, such as *Arabidopsis*, strawberry, tomato, cassava, and barley (Desveaux et al., 2005; Janack et al., 2016; Yan et al., 2020; Hu and Shu, 2021), however, a comprehensive genome-wide analysis of *Whirly* genes in Triticeae species has not been investigated. In this study, a genome-wide analysis of *Whirly* genes was performed in Triticeae species including *Triticum aestivum*, *Triticum urartu*, *Triticum dicoccoides*, *Aegilops tauschii*, *Hordeum vulgare*, and *Secale cereale* to characterize their sequences, gene structures, evolutionary relationships, expression patterns, and stress tolerance under osmotic stress. These results will provide a valuable foundation for further functional investigations of *Whirly* genes in response to osmotic stress.

## Materials and methods

### Plant material and growth conditions

Bread wheat cv. Chinese Spring preserved in our laboratory was used in this study, and the sterilized bread wheat seeds were soaked with ddH_2_O in dark and 4°C condition overnight, then cultured on filter paper wetted with ddH_2_O in a culture room at 25/18°C with 16-h light/8-h dark for 1 week. Next, 7-day-old bread wheat seedlings with uniform leaf size and root length were selected for subsequent experiments. For drought and salt stress treatments, 7-day-old bread wheat seedlings were cultured under 20% PEG6000 (w/v) and 300 mM NaCl treatments, respectively. In each treatment, the root, leaf sheath, and leaf tissues were collected at 0 h, 1 h, and 6 h, then frozen in liquid nitrogen and stored at −80°C for further investigation.

### Genome-wide identification of *Whirly* gene family

The protein sequences of *Triticum aestivum* (Chinese Spring, IWGSC.52), *Triticum urartu* (Tu 2.0), *Triticum dicoccoides* (WEWSeq_v1.0), *Aegilops tauschii* (Aet_v4.0), *Hordeum vulgare* (IBSC_v2), *Brachypodium distachyon* (IBI_v3.0), *Oryza sativa* (Japonica, IRGSP 1.0), *Zea mays* (B73 RefGen_v4), *Solanum lycopersicum* (SL3.0), and *Arabidopsis thaliana* (TAIR10) were downloaded from EnsemblPlants database (http://plants.ensembl.org/index.html). The protein sequence data of *Secale cereale* (Weining v1) was acquired from the China National Center for Bioinformation (CNCB-NGDC Members and Partners, 2022). To identify candidate Whirly protein sequences, the Hidden Markov Model (HMM) profile of the typical Whirly transcription factor domain (PF08536) (Mistry et al., 2021) was used as a query to search against the protein sequences of these 11 plant species with TBtools software (Chen et al., 2020a). Next, the Pfam (https://www.ebi.ac.uk/interpro/) (Mistry et al., 2021) and SMART (Simple Modular Architecture Research Tool, http://smart.embl.de/) (Letunic et al., 2021) online services were used to further confirm the putative Whirly proteins. The protein length, molecular weight, isoelectric point (*p*I), and grand average of hydropathy (GRAVY) of the Whirly proteins were analyzed by WheatOmics 1.0 (Ma et al., 2021).

### Multiple sequence alignment and phylogenetic tree construction

Multiple sequence alignment of Whirly amino acid sequences was performed with ClustalW using the default options in MEGA 11 (Tamura et al., 2021) and visualized by ESPript 3.0 (Gouet et al., 2003). The phylogenetic tree was constructed by using the neighbor-joining (NJ) method with 1,000 bootstrap replicates in MEGA 11 software (Tamura et al., 2021) and visualized by Evolview service (Subramanian et al., 2019).

### Gene structure, conserved motif, domain, and 3D structure analyses

The exon–intron structures of *Whirly* genes were analyzed based on TGT (Triticeae-Gene Tribe) (Chen et al., 2020b). The conserved motifs and domains of Whirly family proteins were annotated using the MEME program (Bailey et al., 2009) and SMART website (Letunic et al., 2021) and visualized by TBtools (Chen et al., 2020a). The Swiss-Model program was used to predict the three-dimensional (3D) structure of Whirly proteins (Waterhouse et al., 2018).

### Chromosome localization, gene duplication, and micro-collinearity analysis

The chromosome localization, micro-collinearity, and paralogous/orthologous gene pairs of *Whirly* genes were identified by using Triticeae-Gene Tribe (TGT) (Chen et al., 2020b). The gene duplication events were determined by Multiple Collinear Scanning Toolkits (MCScanX) (Wang et al., 2012). TBtools was used to calculate the nonsynonymous rate (*K_a_*), synonymous rate (*K_s_*), and the nonsynonymous and synonymous substitution ratio (*K_a_*/*K_s_*) values of the paralogous gene pair with the Nei–Gojobori (NG) method (Chen et al., 2020a).

### Regulatory network analysis

The upstream transcription factors and downstream target genes of *TaWHYs* were predicted by using the wheat integrative gene regulatory network (wGRN) (Chen et al., 2023). Protein–protein interactions (PPIs) were analyzed using the STRING database (Von Mering et al., 2003) and WheatOmics 1.0 (Ma et al., 2021).

### Gene expression analysis by RNA-seq data

To investigate the gene expression patterns in bread wheat under drought stress, bread wheat cv. Chinese Spring was planted in a growth chamber under a photoperiod of 16 h/8 h (light/dark). For drought stress, the seedlings were subjected to water-deficit condition during the seedling stage. The leaf tissues were harvested after 0 days, 2 days, 6 days, and 10 days of treatment, and the total RNA of all the collected samples was extracted. A Nanodrop2000 spectrophotometer was used to determine the quantity and quality of the RNA. A total of 12 bread wheat samples (three biological replicates were conducted for each treatment) were sequenced at Majorbio Bio-Pharm Technology Co. Ltd. (Shanghai, China), and paired-end sequencing was performed with an Illumina Novaseq 6000. The transcriptome data have been submitted to NCBI (BioProject ID: PRJNA1003680).

The transcriptome data of different bread wheat tissues (root and shoot) were obtained from NCBI SRA (DRX002485, DRX002486, DRX002487, DRX002491, DRX002492, and DRX002493). The transcriptome data SRX9781249, SRX9781250, SRX9781251, SRX9781252, SRX9781253, SRX9781254, SRX9781255, SRX9781256, SRX9781257, SRX9781258, SRX9781259, and SRX9781260 were used to analyze the gene expression profiles under NaCl stress in leaves during bread wheat seedling stage.

### RNA extraction and real-time PCR

Real-time PCR was performed to detect the expression pattern of *Whirly* genes according to a previous study (Liu et al., 2022). The total RNA was isolated using RNApure Plant Kit (CWBIO), and the first-strand cDNA was synthesized from 1 μg of total RNA using Prescript III RT ProMix (CISTRO). The real-time PCR was performed using gene-specific primers (**Supplementary Table S1**) with 2× Ultra SYBR Green qPCR Mix (CISTRO), and the *TaActin* gene was selected as a reference control. The real-time PCR cycling parameters were 95°C for 30 s, followed by 45 cycles at 95°C for 5 s and 60°C for 30 s, with a melting curve analysis. All reactions were performed on three technical and biological replicates. The relative expression levels of target genes were calculated using the 2^−△△CT^ method (Livak and Schmittgen, 2001).

### Stress tolerance assay in yeast cells

The coding sequences (CDS) of *Whirly* genes were cloned into a pGADT7 vector using the ClonExpress II One Step Cloning Kit (Vazyme, Nanjing), then transformed into *Saccharomyces cerevisiae* (*S. cerevisiae*) BY4741 or its stress-sensitive mutant BY4741 (Δ*hog1*). The primers are shown in **Supplementary Table S1**. For osmotic and oxidative stress, the yeast cells Δ*hog1* carrying the recombinant vector pGADT7-*TaWHY2-6A*/*TaWHY2-6B*/*TaWHY2-6D*/*TaWHY1-7A*/*TaWHY1-7D* were cultured in YPD liquid medium (1% yeast extract, 2% peptone, and 2% glucose) at 30°C until density reached an OD_600_ of 1.0, then serially diluted (10^−1^, 10^−2^, 10^−3^, 10^−4^) with ddH_2_O. The cells were spotted onto YPD medium plates (1% yeast extract, 2% peptone, 2% glucose, and 2% agar) containing 1.2 M d-sorbitol, 0.4 M NaCl, or 4.0 mM H_2_O_2_ and cultured at 30°C for 3–5 days. The wild-type yeast cells BY4741 and stress-sensitive mutant Δ*hog1* carrying the empty vector pGADT7 were used as positive and negative controls, respectively.

### Drought and salt tolerance assay in *Arabidopsis*

The coding sequences of *TaWHY1-7D* were cloned into the pCAMBIA3301-GFP vector, then transformed into *Agrobacterium tumefaciens* EHA105, and generated *35S:TaWHY1-7D* transgenic *Arabidopsis* lines via the floral dip method. The primers are shown in **Supplementary Table S1**. The transgenic *Arabidopsis* lines were selected via spraying 0.5‰ Basta solution. For drought and salt tolerance assays, WT and *35S:TaWHY1-7D* transgenic *Arabidopsis* were treated with drought (water-deficit) and 500 Mm NaCl conditions.

## Results

### Genome-wide identification and phylogenetic relationship analysis of *Whirly* genes

**Figure 1:**
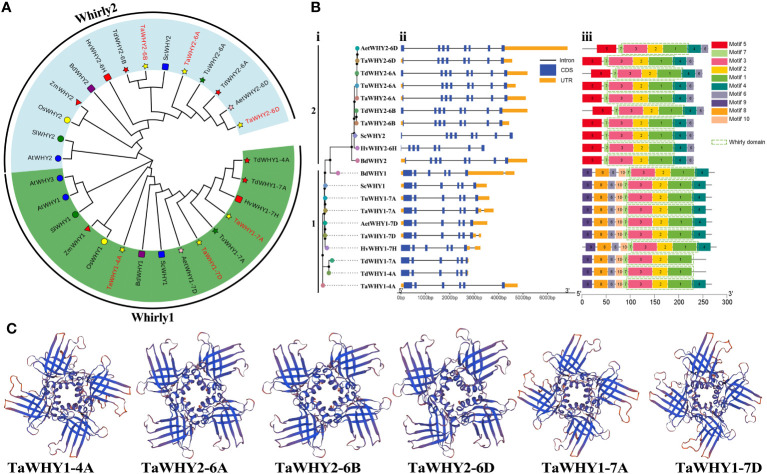
The neighbor-joining (NJ) phylogenetic tree **(A)**, gene structures **(B)**, and 3D structures **(C)** of Whirly proteins. **(A)** The tree was constructed using Whirly protein sequences from *T. aestivum* (Ta), *T. urartu* (Tu), *T. dicoccoides* (Td), *Ae. tauschii* (Aet), *H. vulgare* (Hv) and *S. cereal* (Sc), *B. distachyon* (Bd), *O. sativa* (Os) and *Z. mays* (Zm), *S. lycopersicum* (Sl), and *A*. *thaliana* (At) with bootstrap values of 1,000 replicates. Different groups of Whirly proteins are marked by different colors. **(B)** Phylogenetic classification (i), exon–intron structure (ii), and conserved domain (iii) analyses of *Whirly* genes in Triticeae species. **(C)** The Swiss Model program was used to predict the three-dimensional (3D) structure of the Whirly proteins.

A total of 29 *Whirly* genes were identified from the protein sequences of 11 plant species via a hidden Markov model (HMM) search. In total, 24 *Whirly* genes were identified from nine monocotyledon species, comprising six Triticeae species (*T. aestivum* (6), *T. urartu* (2), *T. dicoccoides* (4), *Ae. tauschii* (2), *H. vulgare* (2), and *S. cereal* (2)) and three other monocotyledon species (*B. distachyon* (2), *O. sativa* (2), and *Z. mays* (2)), while five *Whirly* genes were identified in two dicotyledon species, including *S. lycopersicum* (2) and *A. thaliana* (3) (**Figure 1A**; **Supplementary Table S2**). To further confirm the reliability of the identified *Whirly* genes, the expression of *Whirly* genes was analyzed in *T. urartu*, *T. dicoccoides*, *S. cereale*, *H. vulgare*, and *T. aestivum* based on previous published transcriptomic data (**Supplementary Table S3**). The length of the identified 29 Whirly proteins varied from 223 (*HvWHY2-6H*) to 286 (*ZmWHY1*) amino acid residues, with the molecular weights ranging from 24.24 to 31.71 kDa. The *p*I values ranged from 8.84 (*TdWHY1-4A*) to 10.81 (*SlWHY2*), with the calculated grand average of hydrophilic index (GRAVY) varying from −0.207 (*AtWHY1*) to −0.459 (*TaWHY1-4A*), suggesting that these 29 *Whirly* genes encoded highly hydrophilic proteins (**Supplementary Table S2**).

To elucidate the evolutionary relationship of *Whirly* genes, a phylogenetic tree was constructed using these 29 Whirly proteins (**Figure 1A**). According to the results, *Whirly* genes were classified into two categories, named group 1 (Whirly1) and group 2 (Whirly2). Bread wheat *T. aestivum* (AABBDD, hexaploid) has undergone two rounds of natural hybridization events (Levy and Feldman, 2022). Thus, the number of gene family members in *T. aestivum* (AABBDD) is approximately 1.5- and 3-fold than that in *T. dicoccoides* (AABB, tetraploid) and other diploid Triticeae species, respectively. Consistently, three *Whirly1* or *Whirly2* genes were found in *T. aestivum*, while *T. dicoccoides* and other diploid Triticeae included two and one *Whirly1* or *Whirly2* gene, respectively (**Figure 1A**; **Supplementary Table S2**).

### Gene structure and conserved motif analysis

To investigate the functional divergence of *Whirly* genes, the exon–intron structures, conserved motifs, and 3D structures of *Whirly* genes were analyzed in Triticeae species (**Figure 1**; **Supplementary Figure S1**). The results revealed that *Whirly1* and *Whirly2* genes contained six and eight exons in the Triticeae species, respectively. The conserved motif analysis showed that all Whirly proteins contained the Whirly transcription factor domain (PF08536), which consisted of motifs 1, 2, 3, and 7. These also confirmed the reliability of the identified *Whirly* gene family members. Motif 3 contained the conserved “KGKAAL” sequence, which participated in binding to ssDNA (**Supplementary Figure S1**) (Desveaux et al., 2002; Cappadocia et al., 2012). Almost all Whirly proteins contained motif 4, except for TdWHY1-4A and TdWHY1-7A, which lacked a portion of the amino acid sequences of motif 4 (**Supplementary Figure S1**). Motifs 8, 9, and 10 were present in group 1 members, while they were absent in group 2 members. Motif 5 was unique to group 2 members. In addition, all TaWHY proteins contained two anti-parallel four-stranded *β*-sheets that extend like blades from an *α*-helical core (**Figure 1C**), which were consistent with its “whirligig” structure (Desveaux et al., 2005).

### Chromosomal location, collinearity, and *K_a_*/*K_s_* analysis of *Whirly* genes

**Figure 2:**
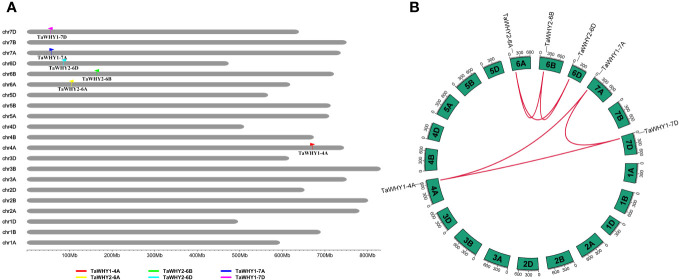
Chromosomal localizations **(A)** and syntenic relationships **(B)** among *TaWHY* genes in *T. aestivum*. **(B)** Red lines in the highlight indicate the syntenic *TaWHY* gene pairs.

The distribution of *Whirly* genes on the chromosome in six Triticeae species (*T. aestivum*, *T. urartu*, *T. dicoccoides*, *Ae. tauschii*, *H. vulgare*, and *S. cereal*), three other monocotyledon species (*B. distachyon*, *O. sativa*, and *Z. mays*), and two dicotyledon species (*S. lycopersicum* and *A. thaliana*) are shown in **Supplementary Table S2**. In *T. aestivum* (AABBDD, hexaploid), *Whirly1* genes were distributed on chromosomes 4A (*TaWHY1-4A*), 7A (*TaWHY1-7A*), and 7D (*TaWHY1-7D*). *Whirly2* genes had three copies in its subgenomes A, B, and D, i.e., *TaWHY2-6A*, *TaWHY2-6B*, and *TaWHY2-6D* were distributed on chromosomes 6A, 6B, and 6D, respectively (**Figure 2**). Similarly, *TdWHY1-4A*, *TdWHY2-6A*, *TdWHY2-6B*, and *TdWHY1-7A* were located on chromosomes 4A, 6A, 6B, and 7A in *T. dicoccoides* (AABB, tetraploid), respectively. *AetWHY2-6D* and *AetWHY1-7D* were distributed on chromosomes 6D and 7D in *Ae. tauschii* (DD, diploid), respectively. *TuWHY2-6A* and *TuWHY1-7A* were located on chromosomes 6A and 7A in *T. urartu* (AA, diploid), respectively. *HvWHY2-6H* and *HvWHY1-7H* were located on chromosomes 6H and 7H in *H. vulgare* (HH, diploid), respectively. In *S*. *cereale* (RR, diploid), *ScWHY1* and *ScWHY2* were distributed on chromosomes 1R and 6R, respectively. Interestingly, the orthologous genes of *TaWHY1-4A* were not distributed on chromosomes 4A in *T. urartu* and 4H in *H. vulgare*, whereas *TdWHY1-4A* existed on chromosome 4A of *T. dicoccoides* (**Supplementary Table S2**). This result suggested that the expansion events of *Whirly* genes occurred through hybridization and polyploidization.

**Figure 3:**
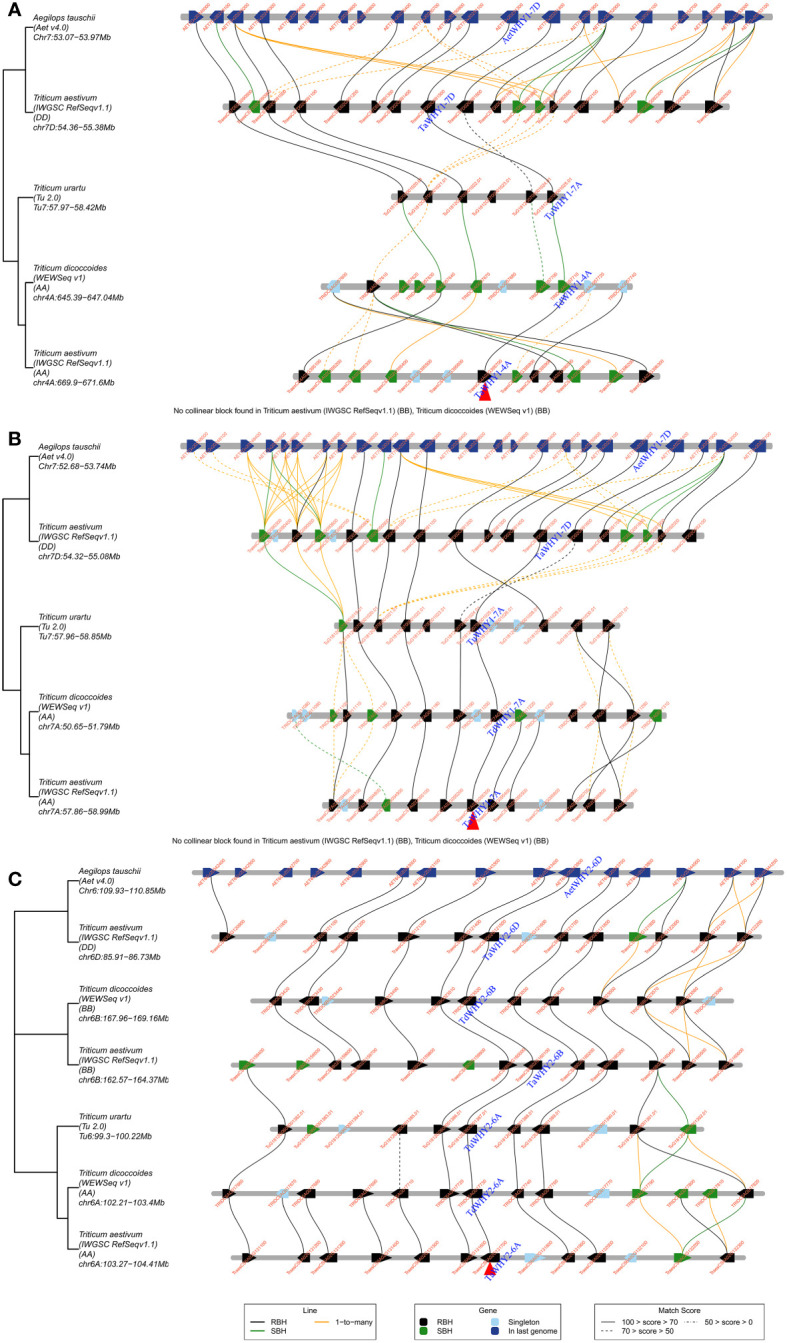
Micro-collinearity analysis of the *Whirly* gene in Triticeae species. *TaWHY1-4A* **(A)**, *TaWHY1-7A* **(B)**, and *TaWHY2-6A* **(C)** were used as the query gene, respectively.

To further investigate the evolutionary process of *TaWHYs*, gene duplication, and micro-collinearity analyses of the *Whirly* genes were performed (**Figure 3**; **Supplementary Table S4**). A total of six paralogous gene pairs of *TaWHYs* (*TaWHY1-4A*/*TaWHY1-7A*/*TaWHY1-7D*, and *TaWHY2-6A*/*TaWHY2-6B*/*TaWHY2-6D*) were identified in bread wheat genome and expanded by whole-genome duplication (WGD) or segmental duplication events (**Figure 2B**; **Supplementary Table S4**). The *K_a_*/*K_s_* values of paralogous gene pairs were all less than 1, indicating that *TaWHY* genes underwent purifying selection to avoid functional divergence (**Supplementary Table S4**). Micro-collinearity analysis contributes to the investigation of the inheritance and variation of specific genes in local regions and detecting the origin of specific genes during the hybridization and polyploidization process (Chen et al., 2020b). To explore the origin of *Whirly* genes in Triticeae species, *TaWHY1-4A*, *TaWHY2-6A*, and *TaWHY1-7A* were used as query genes to analyze the micro-collinearity by TGT (**Figure 3**). The homologous genes of *TaWHY2-6A* were detected in the collinearity regions of *T. urartu*, *Ae. tauschii*, subgenomes A and B of *T. dicoccoides*, and subgenomes B and D of *T. aestivum*, suggesting that the *Whirly2* genes and their adjacent genes in the collinearity regions were relatively conserved during evolutionary processes in Triticeae species. However, no homologous genes of *TaWHY1-4A* and *TaWHY1-7A* were found in the collinearity regions of subgenome B of *T. dicoccoides*, and subgenome B of *T. aestivum*. In addition, the homologous genes of *TaWHY1-4A* were present in the collinearity regions on chromosome 7A of *T. urartu*, and 7D of *Ae. tauschii*, and 7D of *T. aestivum*, but absent on chromosome 4 of *T. urartu*, suggesting that *TaWHY1-4A* and *TdWHY1-4A* might originate from *TuWHY1-7A* or *AetWHY1-7D*.

### Expression patterns analysis of *TaWHYs*

**Figure 4:**
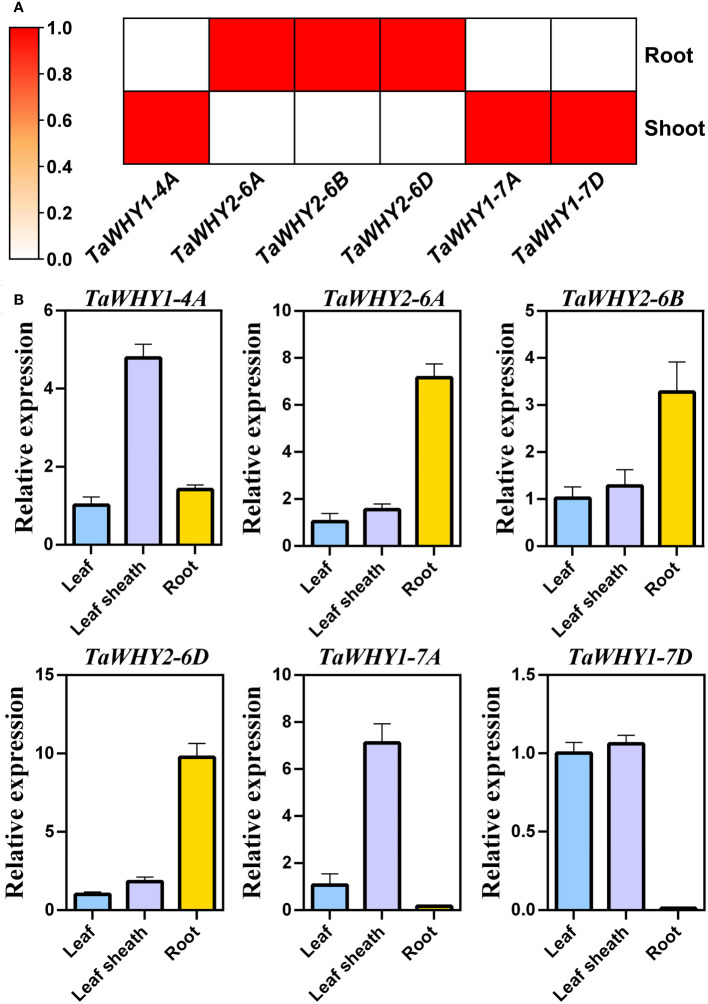
Expression pattern analysis of *TaWHYs* in different tissues. **(A)** The expression levels of *TaWHY* genes in root and shoot were determined through RNA-seq analysis. Fragments per kilobase of exon per million mapped fragments (FPKM) values were used to measure the expression levels of genes. **(B)** The expression levels of *TaWHY* genes in the root, leaf sheath, and leaf during the bread wheat seedling stage were determined by real-time PCR. The expression level of the bread wheat *actin* gene was used as the reference control to standardize the RNA samples for each reaction. Data represent the mean ± SD of three replicates.

**Figure 5:**
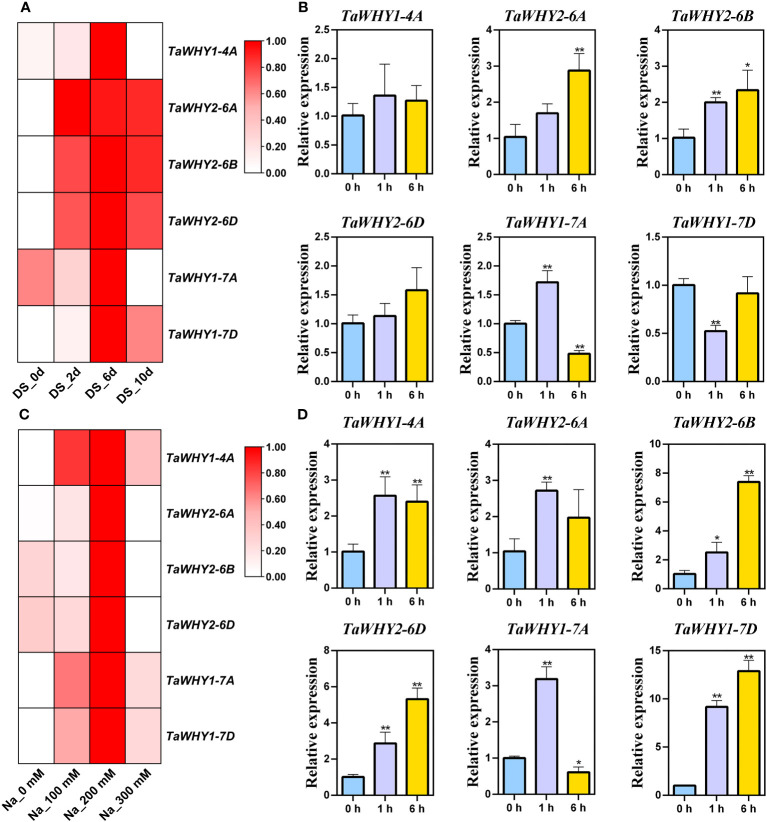
Expression patterns of *TaWHY* genes in response to osmotic stress. **(A)** RNA-seq analysis of the expression profiles of *TaWHY* genes in response to drought stress for 0 days, 2 days, 6 days, and 10 days, respectively. Fragments per kilobase of exon per million mapped fragments (FPKM) values were used to measure the expression levels of genes. **(B)** The expression profiles of *TaWHY* genes in bread wheat seedling leaves at 0 h, 1 h, and 6 h after PEG stress treatment. **(C)** RNA-seq analysis of the expression profiles of *TaWHY* genes in response to 0 mM, 100 mM, 200 mM, and 300 mM NaCl treatment. FPKM values were used to measure the expression levels of genes. **(D)** The expression profiles of *TaWHY* genes in bread wheat seedling leaves at 0 h, 1 h, and 6 h after NaCl stress treatment. The expression level of the bread wheat *actin* gene was used as the reference control to standardize the RNA samples for each reaction. Data represent the mean ± SD of three replicates.  The asterisk indicates significant differences compared with 0 h (control, as 1) based on Student’s *t*-test (^*^ *p* < 0.05; ^**^ *p* < 0.01).

To insight into the biological function of *TaWHY* genes, the transcriptome data and real-time PCR were used to determine the expression patterns of six *TaWHY* genes in different tissues (leaves, leaf sheaths, and roots during bread wheat seedling stage) and in response to osmotic (drought and salt) stress (**Figures 4**, **5**). The analysis of the transcriptome data revealed that the *TaWHY1* genes (*TaWHY1-4A*, *TaWHY1-7A*, and *TaWHY1-7D*) exhibited the highest expression levels in leaves, and the *TaWHY2* genes (*TaWHY2-6A*, *TaWHY2-6B*, and *TaWHY2-6D*) showed the highest expression levels in roots (**Figure 4A**). Consistently, the real-time PCR results showed that *TaWHY1* genes (*TaWHY1-4A*, *TaWHY1-7A*, and *TaWHY1-7D*) were highest expressed in leaf sheaths, followed by leaves, and roots during the bread wheat seedling stage. *TaWHY2* genes (*TaWHY2-6A*, *TaWHY2-6B*, and *TaWHY2-6D*) exhibited the highest expression level in roots, followed by leaf sheaths, and finally in leaves (**Figure 4B**).

After drought stress treatment, RNA-seq analysis revealed that the *TaWHY1* genes exhibited the highest expression levels after 6 days of drought treatment, and the expression of *TaWHY2* genes increased with the progression of drought stress duration (**Figure 5A**). The real-time PCR results demonstrated the expression of *TaWHY1-7A* was up-regulated under PEG stress, peaking at 1 h with 1.6-fold compared with the control, *TaWHY1-7D* was down-regulated, and *TaWHY1-4A* was not significantly changed. The expression of *TaWHY2-6A*, *TaWHY2-6B*, and *TaWHY2-6D* (group 2) was gradually up-regulated and reached the highest expression level at 6 h under PEG stress with approximately 2.9-, 2.3-, and 1.6-fold compared with the control, respectively (**Figure 5B**).

After NaCl treatment, the expression levels of *TaWHY* genes were significantly up-regulated (**Figures 5C, D**). The real-time PCR results revealed that the expression levels of *TaWHY1-4A*, *TaWHY2-6A*, *TaWHY2-6B*, *TaWHY2-6D*, *TaWHY1-7A*, and *TaWHY1-7D* were all increased, peaking at 1 h, 1 h, 6 h, 6 h, 1 h, and 6 h with approximately 2.6-, 2.7-, 7.4-, 2.9-, 3.2-, and 12.9-fold compared with the control, respectively (**Figure 5D**). Therefore, we speculated that *TaWHYs* might play an important role under osmotic stress.

### Upstream transcription factors, downstream target genes, and interacting proteins analysis of TaWHYs

**Figure 6:**
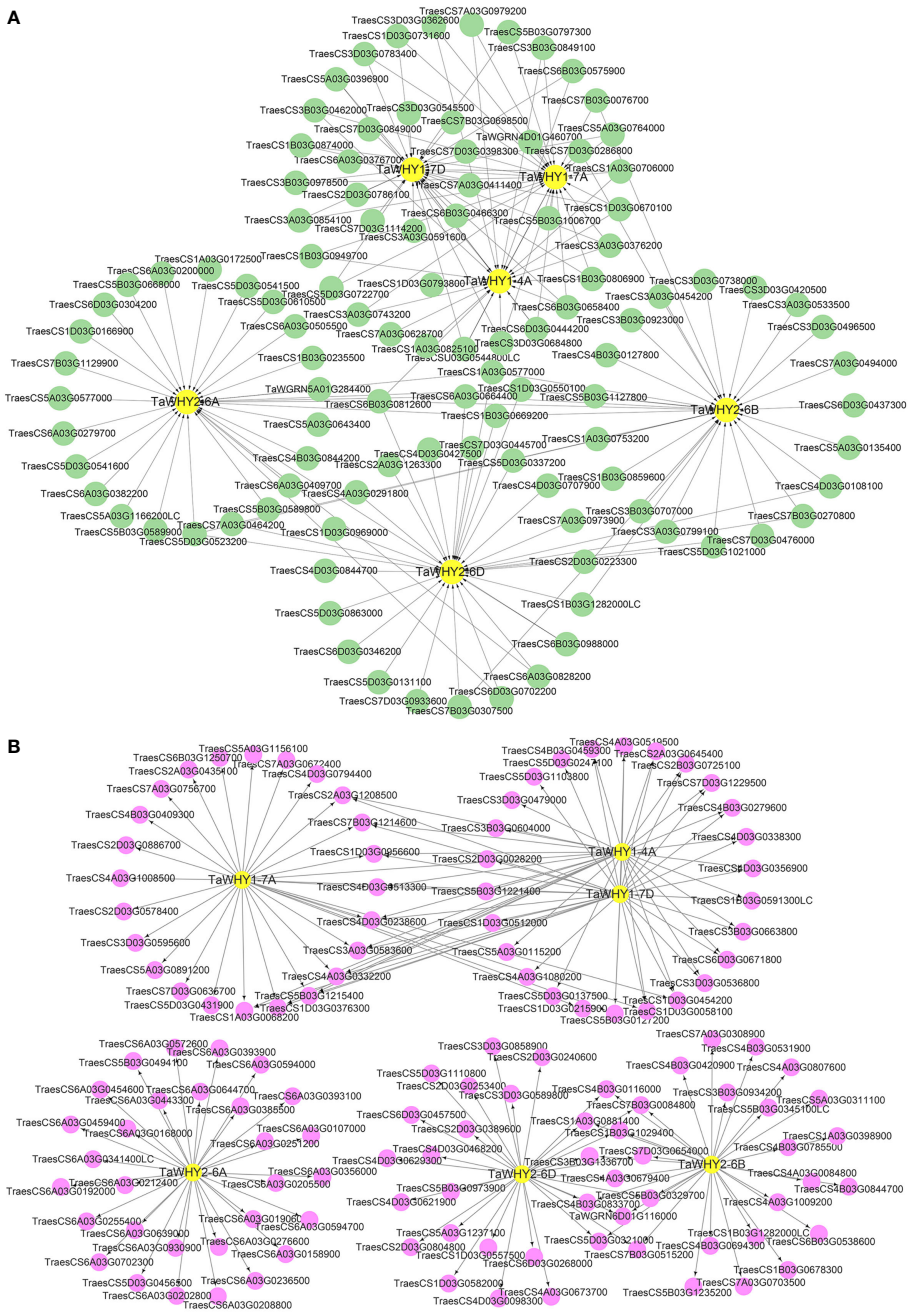
The upstream transcription factor **(A)** and downstream target gene **(B)** analyses of *TaWHY* genes.

Transcription factors can interact with different *cis*-elements in the promoter region of target genes, exerting diverse functions in plant growth, development, and stress response (Strader et al., 2022). To determine the functions of *TaWHY* genes, upstream transcription factors and downstream target genes of *TaWHYs* were predicted by using the wheat integrative gene regulatory network (wGRN) (**Figure 6**; **Supplementary Table S5**) (Chen et al., 2023). Then, 22, 28, 33, 44, 195, and 187 transcription factors were predicted to regulate the expression of *TaWHY1-4A*, *TaWHY1-7A*, *TaWHY1-7D*, *TaWHY2-6A*, *TaWHY2-6B*, and *TaWHY2-6D*, respectively (**Supplementary Table S5**). We also conducted an analysis of the expression patterns for the top 30 potential upstream transcription factors and downstream target genes associated with *TaWHYs*. Under drought stress, the expression patterns of the cytokinin-responsiveGATA transcription factor 1-like gene (*TraesCS6B03G0575900*) were most similar to *TaWHY1-4A*. Additionally, the most similar expression patterns were observed in the transcription factor GLK2 (*TraesCS3D03G0362600*) and TCP family transcription factor TCP5 (*TraesCS3A03G0743200*, *TraesCS3B03G0849100*) with *TaWHY1-7A*. The MYB transcription factor (*TraesCS6B03G0466300*) and the cytokinin-responsive GATA transcription factor 1-like gene (*TraesCS6B03G0575900*) exhibited the most similar expression patterns to *TaWHY1-7D*. Furthermore, the nuclear transcription factor Y subunit C-4-like (*TraesCS6A03G0382200*) showed the most similar expression patterns to *TaWHY2-6A*. The transcription factor bHLH49-like gene (*TraesCS4D03G0108100*) demonstrated the most similar expression patterns to *TaWHY2-6B* and *TaWHY2-6D* (**Figure 6A**; **Supplementary Figure S2A**). Under salt stress, transcription factors LSD1 (*TraesCS1A03G0706000* and *TraesCS1B03G0806900*) and GLK2 (*TraesCS3A03G0376200*) exhibited the most similar expression patterns to *TaWHY1-4A*. The transcription factors GLK2 (*TraesCS3A03G0376200*), LSD1 (*TraesCS1A03G0706000*), GATA transcription factor 17-like (*TraesCS6A03G0279700*), RAP2-9-like (*TraesCS7B03G0076700*), and Zinc finger CCCH domain-containing protein 44-like (*TraesCS7A03G0973900*) displayed the most similar expression patterns to *TaWHY1-7A*, *TaWHY1-7D*, *TaWHY2-6A*, *TaWHY2-6B*, and *TaWHY2-6D*, respectively (**Figure 6A**; **Supplementary Figure S2B**). These transcription factors are highly likely to regulate the expression of *TaWHY* genes under drought and salt stress.

TaWHYs, as transcription factors, also regulate downstream target genes in response to osmotic stress. The result suggested that *TaWHY1-4A*, *TaWHY1-7A*, *TaWHY1-7D*, *TaWHY2-6A*, *TaWHY2-6B*, and *TaWHY2-6D* might bind to the promoter of 1,345, 1,181, 1,404, 999, 3,413, and 3,662 downstream target genes, respectively (**Supplementary Table S6**). Under drought stress, the similar expression patterns were observed in fructokinase-like 1 (*TraesCS3A03G0869600*), protein fluorescent in blue light (*TraesCS5D03G0431900*), 2-carboxy-1,4-naphthoquinone phytyltransferase (*TraesCS4A03G1008500*), 50S ribosomal protein (*TraesCS4A03G0332200* and *TraesCS6B03G1250700*), and starch synthase (*TraesCS4D03G0513300*) with *TaWHY1-7A*. The gene of glutamyl-tRNA (Gln) amidotransferase (*TraesCS2A03G0645400*), CDK5RAP1-like protein (*TraesCS4D03G0338300*), chaperone protein dnaJ 6-like (*TraesCS6A03G0385500*), OSB (*TraesCS3B03G1336700*), and flap endonuclease (*TraesCS1B03G1029400*) exhibited the most similar expression patterns to *TaWHY1-4A*, *TaWHY1-7D*, *TaWHY2-6A*, *TaWHY2-6B*, and *TaWHY2-6D*, respectively (**Figure 6B**; **Supplementary Figure S3A**). Similarly, the downstream target genes of the most similar expression patterns with *TaWHYs* under salt stress were also detected, i.e., *TaWHY1-4A* with transcription termination/antitermination protein NusG-like (*TraesCS5B03G1215400*), *TaWHY1-7A* with protein fluorescent in blue light (*TraesCS5D03G0431900*), *TaWHY1-7D* with superoxide dismutase (*TraesCS4A03G1080200*) and transcription termination/antitermination protein NusG-like (*TraesCS5B03G1215400*), *TaWHY2-6A* with eukaryotic translation initiation factor 3 subunit F-like (T*raesCS6A03G0205500*), *TaWHY2-6B* with HSP20-like chaperones superfamily protein (*TraesCS7D03G0654000*) and eukaryotic translation initiation factor 3 subunit K (*TraesCS4B03G0785500*), and *TaWHY2-6D* with HSP20-like chaperones superfamily protein (*TraesCS7D03G0654000*), DNA polymerase delta small subunit-like (*TraesCS4B03G0833700*) and flap endonuclease 1-A-like (*TraesCS1B03G1029400* and *TraesCS1A03G0881400*) (**Figure 6B**; **Supplementary Figure S3B**). The GO enrichment analysis result showed the downstream target genes of TaWHY1s mainly participated in translation, glutaminyl-tRNAGln biosynthesis, protoporphyrinogen IX biosynthetic process, and heme biosynthetic process (**Supplementary Figure S4**). TaWHY2s might take part in mRNA splicing, RNA binding, and DNA replication (**Supplementary Figure S4**). It was worth noting that TaWHY1-7D and TaWHY2-6D were predicted to respond to hydrogen peroxide (H_2_O_2_) and oxidative stress (**Supplementary Figure S4**), suggesting *TaWHY1-7D* and *TaWHY2-6D* might respond to osmotic stress via regulating ROS homeostasis.

**Figure 7:**
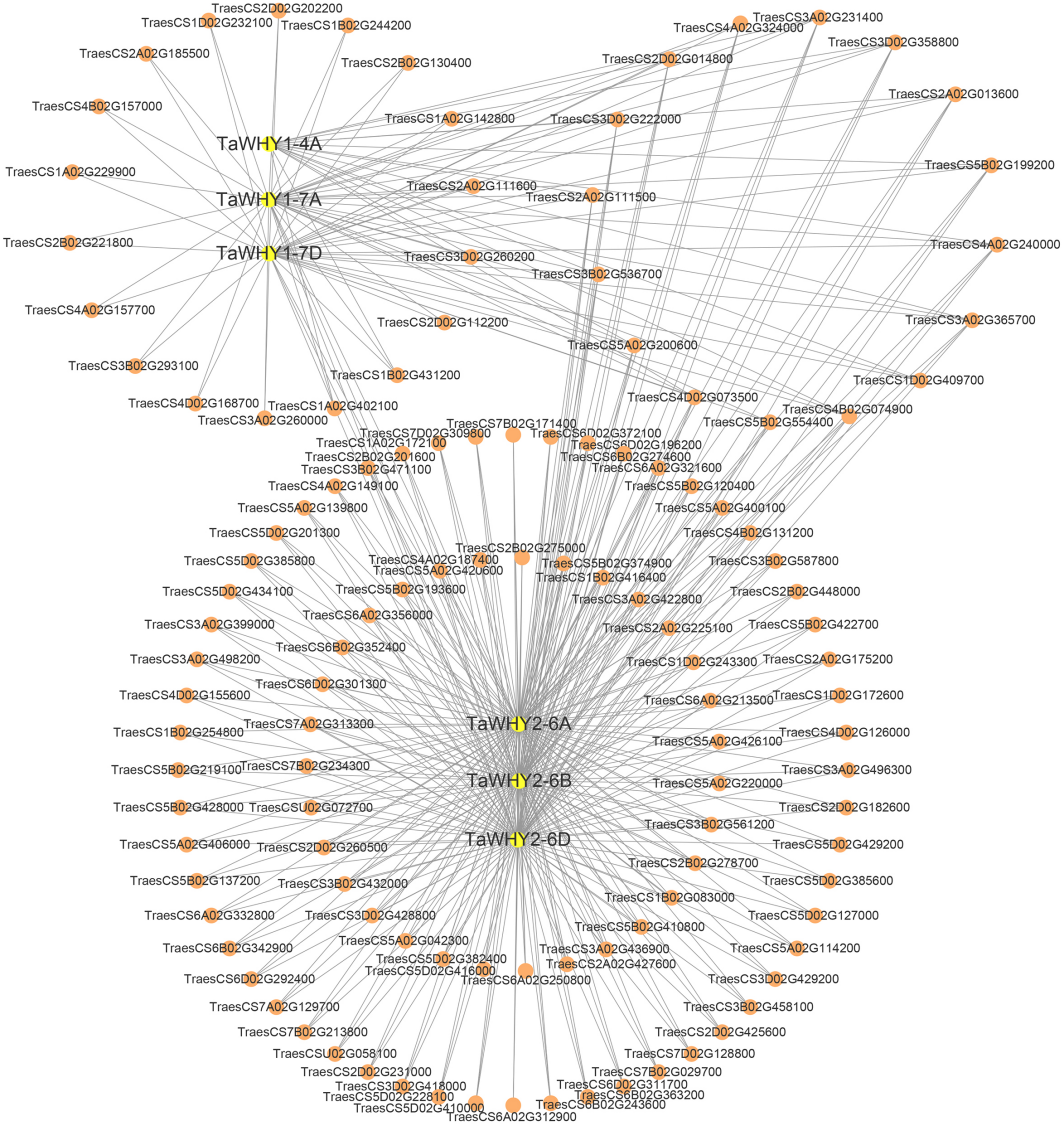
Protein–protein interaction (PPI) network analysis of TaWHY proteins.

The protein–protein interactions (PPIs) analysis suggested that TaWHY1-4A, TaWHY1-7A, and TaWHY1-7D could interact with 16, 37, and 36 proteins, respectively. TaWHY2-6A, TaWHY2-6B, and TaWHY2-6D interact with 102 proteins (**Supplementary Table S7**). We identified the interacting proteins with similar expression patterns to TaWHYs under drought stress (**Supplementary Figure S5**), i.e., TaWHY1-4A was found to interact with fructokinase-like 2 (TraesCS2A02G013600). TaWHY1-7A showed interactions with glutamate-rich WD repeat-containing protein (TraesCS4B02G157000), fructokinase-like 2 (TraesCS2A02G013600), and serine/arginine-rich splicing factor SR34A (TraesCS4D02G168700). TaWHY1-7D demonstrated an interaction with fructokinase-like 2 (TraesCS2A02G013600). Additionally, TaWHY2-6A interacted with DnaJ protein homolog (TraesCS5B02G374900), while TaWHY2-6B and TaWHY2-6D showed interactions with methionine aminopeptidase 1B (TraesCS2B02G448000) and protein OSB2 (TraesCS3B02G536700) (**Figure 7**; **Supplementary Figure S5A**). After NaCl treatment, TaWHY1 (TaWHY1-4A, TaWHY1-7A, and TaWHY1-7D) showed the most similar expression patterns with interacting protein single-stranded DNA-binding protein (TraesCS3A02G231400). TaWHY2 (TaWHY2-6A, TaWHY2-6B, and TaWHY2-6D) demonstrated the most similar expression patterns with glutamate-rich WD repeat-containing protein (TraesCS5B02G137200), actin-related protein (TraesCS5B02G422700), chaperone protein dnaJ A6 (TraesCS6B02G274600), and methionine aminopeptidase 1B (TraesCS2D02G231000) (**Figure 7**; **Supplementary Figure S5B**). These results suggested the regulatory mechanism of *TaWHY* genes to avoid or defend against osmotic stress.

### TaWHYs improve the tolerance to osmotic and oxidative stresses in yeast cells

**Figure 8:**
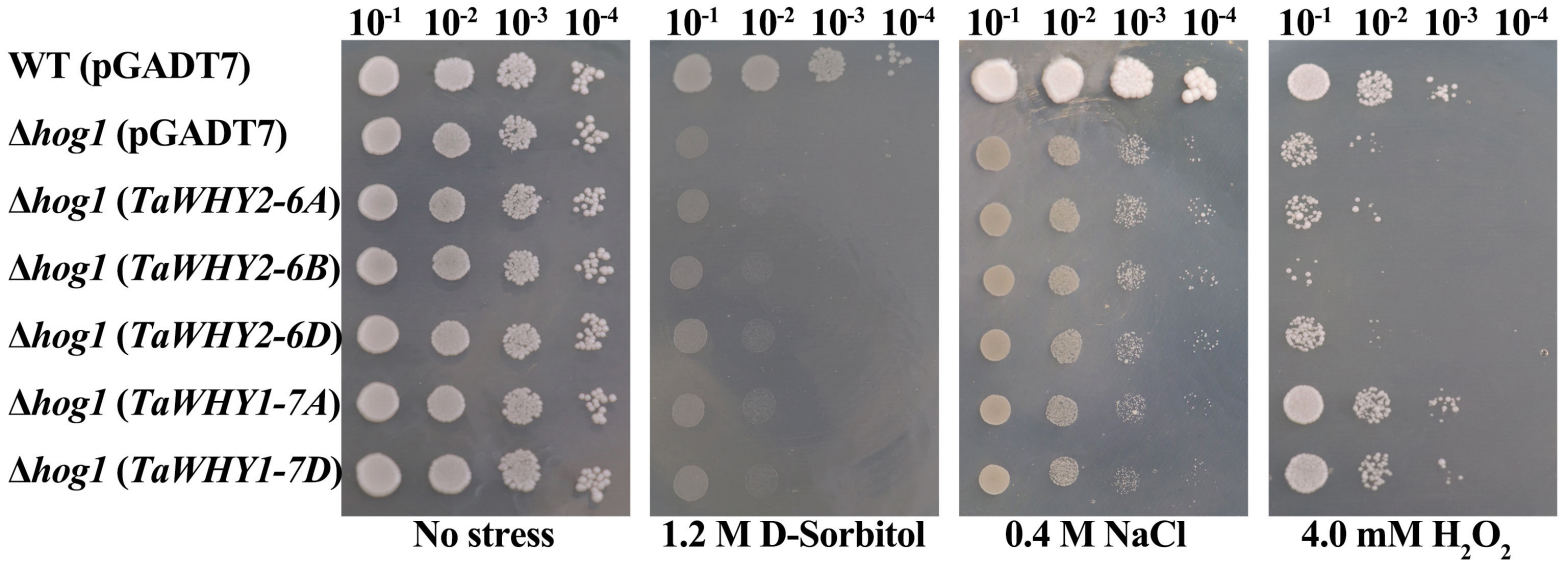
The ability of the tolerance in response to 1.2 M d-sorbitol, 0.4 M NaCl, and 4.0 mM H_2_O_2_ stresses in recombinant yeast cells. For osmotic and oxidative stresses, the yeast cells Δ*hog1* carrying the recombinant vector pGADT7-*TaWHY2-6A*/*TaWHY2-6B*/*TaWHY2-6D*/*TaWHY1-7A*/*TaWHY1-7D* were spotted onto YPD medium plates containing 1.2 M d-sorbitol, 0.4 M NaCl, or 4.0 mM H_2_O_2_ with serially diluted (10^−1^, 10^−2^, 10^−3^, 10^−4^) and cultured at 30°C for 3–5 days. The wild-type yeast cells BY4741 and the stress-sensitive mutant Δ*hog1* carrying the empty vector pGADT7 were used as positive and negative controls, respectively.

To further investigate the function of *TaWHY* genes under osmotic (d-sorbitol and NaCl) and oxidative (H_2_O_2_) stresses, *TaWHY2-6A*, *TaWHY2-6B*, *TaWHY2-6D*, *TaWHY1-7A*, and *TaWHY1-7D* were cloned into the pGADT7 vector, and then transformed into the yeast cells BY4741 or stress-sensitive yeast mutant BY4741 (Δ*hog1*) to confirm the ability to improve stress resistance in yeast cells (**Figure 8**). The results suggested that the growth of the BY4741 or Δ*hog1* yeast cells carrying these *TaWHY* genes was not obviously different compared with the control (pGADT7 empty vector) under normal growth conditions. After d-sorbitol treatment, Δ*hog1* yeast cells overexpressing *TaWHYs* slightly enhanced their tolerance to d-sorbitol stress in comparison to the negative control. The Δ*hog1* yeast overexpressing *TaWHY2-6A*, *TaWHY2-6B*, and *TaWHY2-6D* obviously improved the resistance to NaCl stress, but the colonies of Δ*hog1* with *TaWHY1-7A* and *TaWHY1-7D* were slightly increased compared with the negative control under NaCl stress.

Adverse environmental conditions induce ROS production; ROS accumulation can cause oxidative damage to membranes, proteins, and RNA and DNA molecules and even lead to the oxidative destruction of the cell in a process termed oxidative stress; thereby, ROS scavenging is essential for plants to avoid or defend against adverse stress (Choudhury et al., 2017). To determine whether *TaWHYs* enhanced stress tolerance by scavenging ROS in yeast cells, Δ*hog1* yeast cells carrying pGADT7-*TaWHYs* or pGADT7 were grown on YPD medium containing 4.0 mM H_2_O_2_, suggesting *TaWHY1-7A* and *TaWHY1-7D* strongly enhanced the oxidative stress tolerance in yeast, but the colonies of Δ*hog1* overexpressing *TaWHY2-6A*, *TaWHY2-6B*, and *TaWHY2-6D* were reduced compared with control. These results indicated that the *TaWHY1* and *TaWHY2* genes performed diverse functions. *TaWHY1* mainly enhanced the tolerance to oxidative stresses; *TaWHY2* mainly improved NaCl stress tolerance and was sensitive to oxygen stress; and *TaWHY1* and *TaWHY2* genes slightly improved the tolerance to d-sorbitol stress.

### TaWHY1-7D confers drought and salt tolerance in *Arabidopsis*

**Figure 9:**
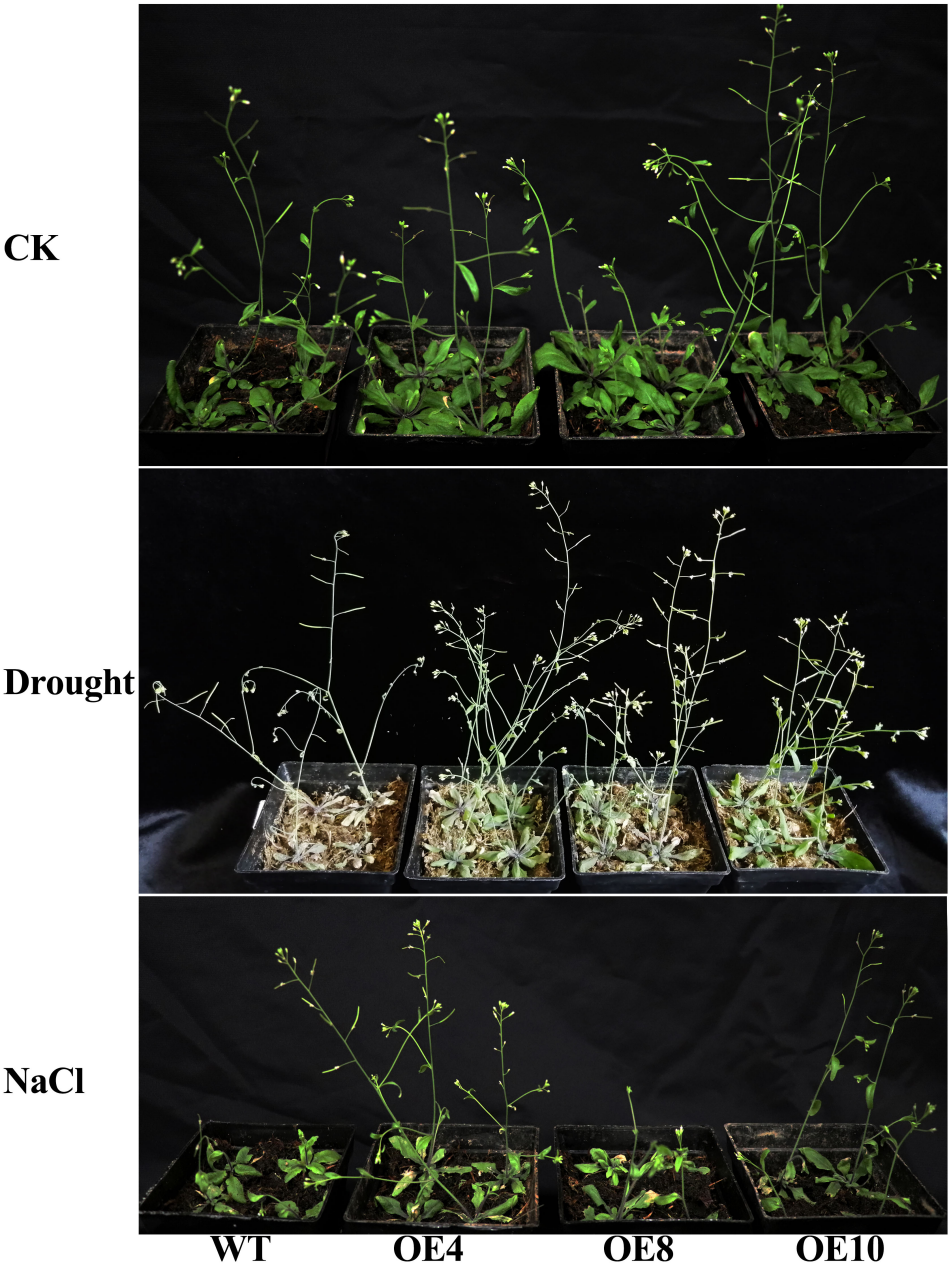
The phenotype of the *35S: TaWHY1-7D* transgenic *Arabidopsis* under drought and NaCl stress. Three independent *35S:TaWHY1-7D* transgenic *Arabidopsis* lines (OE4, OE8, and OE10) and wild type (WT) were chosen for functional analysis of *TaWHY1-7D* under normal conditions (CK), drought (water-deficit), and salt (NaCl) stress treatments.

In order to further confirm the potential role of *TaWHY1-7D* in response to drought and salt stresses, we generated *35S:TaWHY1-7D* transgenic *Arabidopsis* lines. Three independent transgenic lines (OE4, OE8, and OE10) and wild-type (WT) were chosen for the functional analysis of *TaWHY1-7D* in response to drought and salt stresses (**Figure 9**; **Supplementary Figure S6**). The results showed that there were no obvious phenotypic differences between transgenic and WT plants under normal conditions. After an 8-day drought treatment, the wild-type (WT) plants exhibited wilting and subsequent yellowing. In contrast, the transgenic *Arabidopsis* overexpressing *TaWHY1-7D* remained predominantly green. After NaCl treatment for 8 days, both WT and transgenic *Arabidopsis* lines exhibited growth inhibition compared with CK. The growth inhibition was more severe in WT plants compared to transgenic *Arabidopsis*. Thus, the heterologous expression of *TaWHY1-7D* greatly improved drought and salt tolerance in transgenic *Arabidopsis*.

## Discussion

### Evolutionary relationship of *Whirly* genes in Triticeae species

*Whirly* genes have been identified in diverse plant species (Desveaux et al., 2005; Janack et al., 2016; Yan et al., 2020; Hu and Shu, 2021). Most plant species have two kinds of Whirly proteins, Whirly1 and Whirly2, whereas *Arabidopsis* and cassava have three Whirly proteins (Cappadocia et al., 2013; Yan et al., 2020). As a heterologous hexaploid species composed of three subgenomes A, B, and D, bread wheat (AABBDD) has undergone two rounds of natural hybridization events (Levy and Feldman, 2022). Therefore, bread wheat has six *Whirly* genes belonging to *Whirly1* and *Whirly2*, and other Triticeae species, including *T. urartu* (AA, diploid), *T. dicoccoides* (AABB, tetraploid), *Ae. tauschii* (DD, diploid), *H. vulgare* (HH, diploid), and *S. cereal* (RR, diploid), have two, four, two, two, and two *Whirly* genes, respectively (**Figure 1A**; **Supplementary Table S2**). There was a positive correlation between the number of *Whirly* genes and that of subgenomes in Triticeae species.

The paralogous *Whirly* gene pairs *TaWHY1-4A*/*TaWHY1-7A*/*TaWHY1-7D* and *TaWHY2-6A*/*TaWHY2-6B*/*TaWHY2-6D* were identified in *T. aestivum* genome, which all expanded by WGD or segmental duplication events (**Figure 2B**; **Supplementary Table S4**). Interestingly, the paralogous genes of *TaWHY1-7A* and *TaWHY1-7D* were found on chromosome 4A instead of chromosome 7B in *T. aestivum* (**Figure 2B**). To investigate the origin of *TaWHY1-4A*, a micro-collinear analysis of *TaWHY1-4A* was performed. The results showed that the homologous gene of *TaWHY1-4A* did not exist on subgenome B in other related Triticeae species, but there was homologous gene of *TuWHY1-7A* on chromosome 7A of *T. urartu* and *AetWHY1-7D* on chromosome 7D of *Ae. tauschii* (**Figure 3**). Similar events also occurred in the SHMT gene family of *T. aestivum* (Hu et al., 2022). Therefore, we speculated that the expansion events of *Whirly1* genes occurred through hybridization and polyploidization, and *TaWHY1-4A* and *TdWHY1-4A* might have originated from *TuWHY1-7A* or *AetWHY1-7D* (**Figure 3**). However, this speculation still needs further research.

### The function of *TaWHY* genes in response to osmotic stress

Whirly proteins are plant-specific transcription factors that regulate plant development and stress resistance in plants (Krupinska et al., 2022; Taylor et al., 2022). Previous studies mainly focused on the function of *Whirly* genes under abiotic stress and biotic stresses, such as drought (Yan et al., 2020), salt (Akbudak and Filiz, 2019), chilling (Zhuang et al., 2020b) or light stresses (Swida-Barteczka et al., 2018). Previous studies indicated that AtWHY1 located in chloroplasts and nucleus (Krause et al., 2005; Ren et al., 2017) could repress the expression of *WRKY53* and delay leaf senescence in *Arabidopsis* (Miao et al., 2013), whereas AtWHY2 was located in the mitochondria and nucleus (Krause et al., 2005; Golin et al., 2020). These were consistent with the higher expression of *TaWHY1* genes (*TaWHY1-4A*, *TaWHY1-7A*, and *TaWHY1-7D*) in leaf sheaths and leaves and higher expression of *TaWHY2* genes (*TaWHY2-6A*, *TaWHY2-6B*, and *TaWHY2-6D*) in roots (**Figure 4**).

Recently, *Whirly* genes were reported to improve osmotic stress resistance in plants, such as MeWHYs, which could interact with MeCIPK23 to activate ABA biosynthesis and regulate drought resistance in cassava (Yan et al., 2020). In this study, *TaWHY1-7A* and three *TaWHY2* genes were up-regulated under PEG stress, *TaWHY1-7D* was down-regulated, and *TaWHY1-4A* was not significantly changed (**Figure 5**), suggesting that functional differentiation of *Whirly* genes occurred. All *TaWHYs* were up-regulated under NaCl stress (**Figure 5**) and improved the resistance of NaCl stress in yeast, respectively (**Figure 8**). The heterologous expression of *TaWHY1-7D* greatly improved drought and salt tolerance in transgenic *Arabidopsis* (**Figure 9**). In addition, *Whirly* genes have been reported to regulate ROS homeostasis (Lin et al., 2019), and our results also showed that *TaWHY1-7A* and *TaWHY1-7D* strongly enhanced the oxidative stress tolerance in yeast cells (**Figure 8**). ROS scavenging also might be an important reason for the improvement of stress resistance in *TaWHY1* genes. However, the growth of Δ*hog1* overexpressing *TaWHY2-6A*, *TaWHY2-6B*, and *TaWHY2-6D* was inhibited under oxidative stress; these were consistent with a previous study that found that overexpression of *AtWHY2* caused the accumulation of ROS in the plant (Cai et al., 2015). The ROS accumulation might cause cellular stress, thus activating the alternative pathway to reduce ROS levels and eliminate the stress (Cai et al., 2015). GO enrichment analysis also showed that TaWHY1-7D and TaWHY2-6D regulated downstream target genes to respond to H_2_O_2_ and oxidative stress (**Supplementary Figure S4**). Based on the above research, we speculate that the *Whirly* genes may play a vital role in plant resistance to osmotic stress. These results provide useful information for further functional studies of *Whirly* genes and lay a foundation to improve wheat yield and quality via molecular breeding under osmotic stress.

## Data availability statement

The original contributions presented in the study are included in the article/**Supplementary Material**. Further inquiries can be directed to the corresponding authors.

## Author contributions

HL: Conceptualization, Data curation, Formal Analysis, Funding acquisition, Investigation, Methodology, Project administration, Resources, Supervision, Validation, Visualization, Writing – original draft, Writing – review & editing. XW: Writing – review & editing. WY: Writing – review & editing. WL: Writing – review & editing. YW: Resources, Writing – review & editing. QW: Resources, Writing – review & editing. YZ: Resources, Writing – review & editing.

## Supplementary material

Supplementary Figure 1 Multiple sequence alignment of the conserved domains of *Whirly* genes in Triticeae species. Moitf1-10 and DNA binding domain were marked. The conserved cysteine was marked by red triangle.

Supplementary Figure 2 The FPKM values of upstream transcription factors of TaWHYs under drought **(A)** and salt **(B)** stress.

Supplementary Figure 3 The FPKM values of downstream target genes of TaWHYs under drought **(A)** and salt **(B)** stress.

Supplementary Figure 4 GO enrichment analysis on the downstream target genes of *TaWHY* genes.

Supplementary Figure 5 The FPKM values of interacting protein of TaWHYs under drought **(A)** and salt **(B)** stress.

Supplementary Figure 6 The PCR detection **(A)** and screening **(B)** of *35S: TaWHY1-7D* transgenic Arabidopsis.
