# Supplementary figures and images for: Identification of Whirly transcription factors in Triticeae species and functional analysis of TaWHY1-7D in response to osmotic stress
Source: Front Plant Sci. 2023 Dec 5;14:1297228. doi: 10.3389/fpls.2023.1297228 (PMC10728677; doi:10.3389/fpls.2023.1297228)

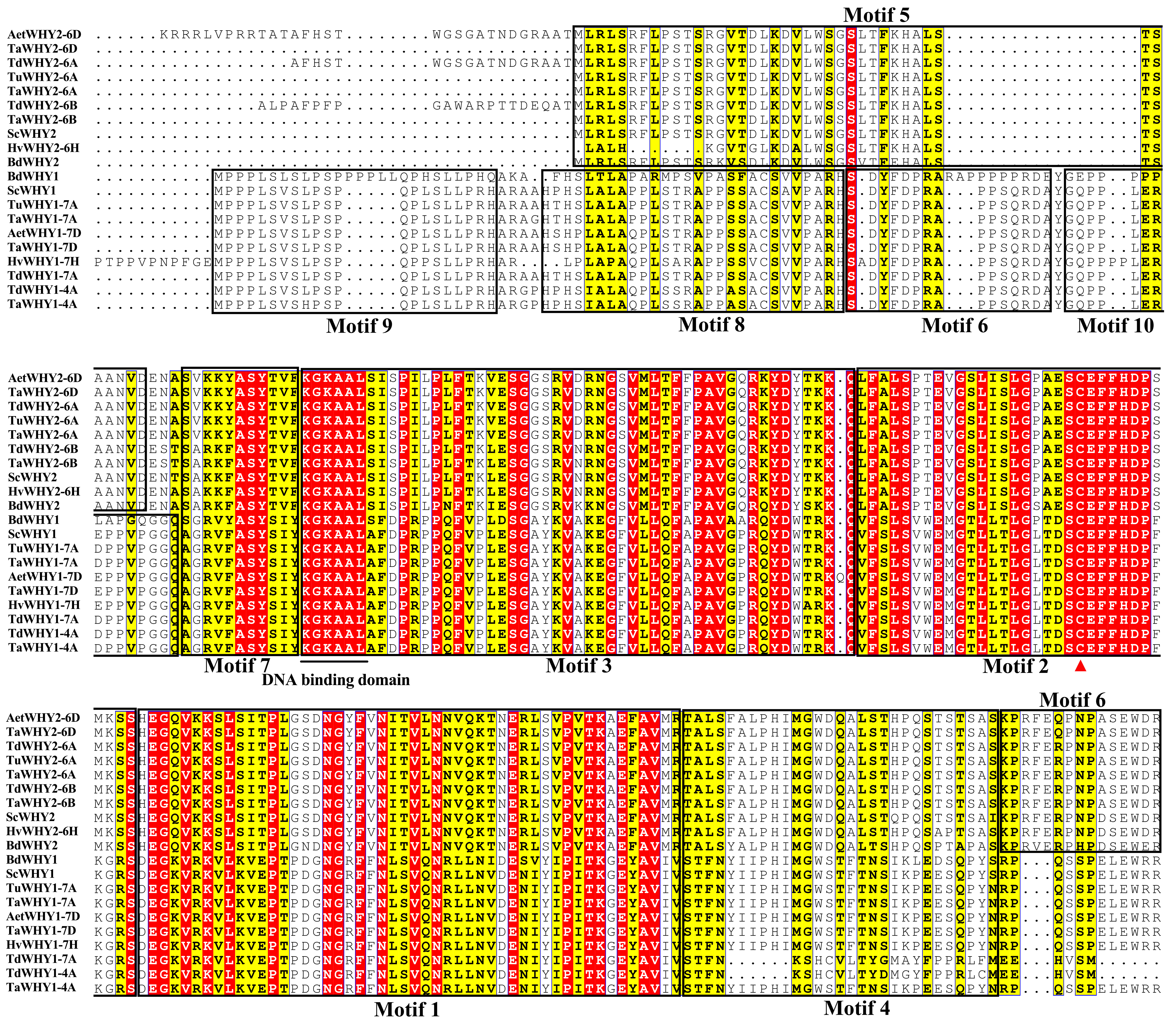

Supplement: Supplementary Figure 1 — Multiple sequence alignment of the conserved domains of Whirly genes in Triticeae species. Moitf1-10 and DNA binding domain were marked. The conserved cysteine was marked by red triangle. [file Image_1.jpeg]

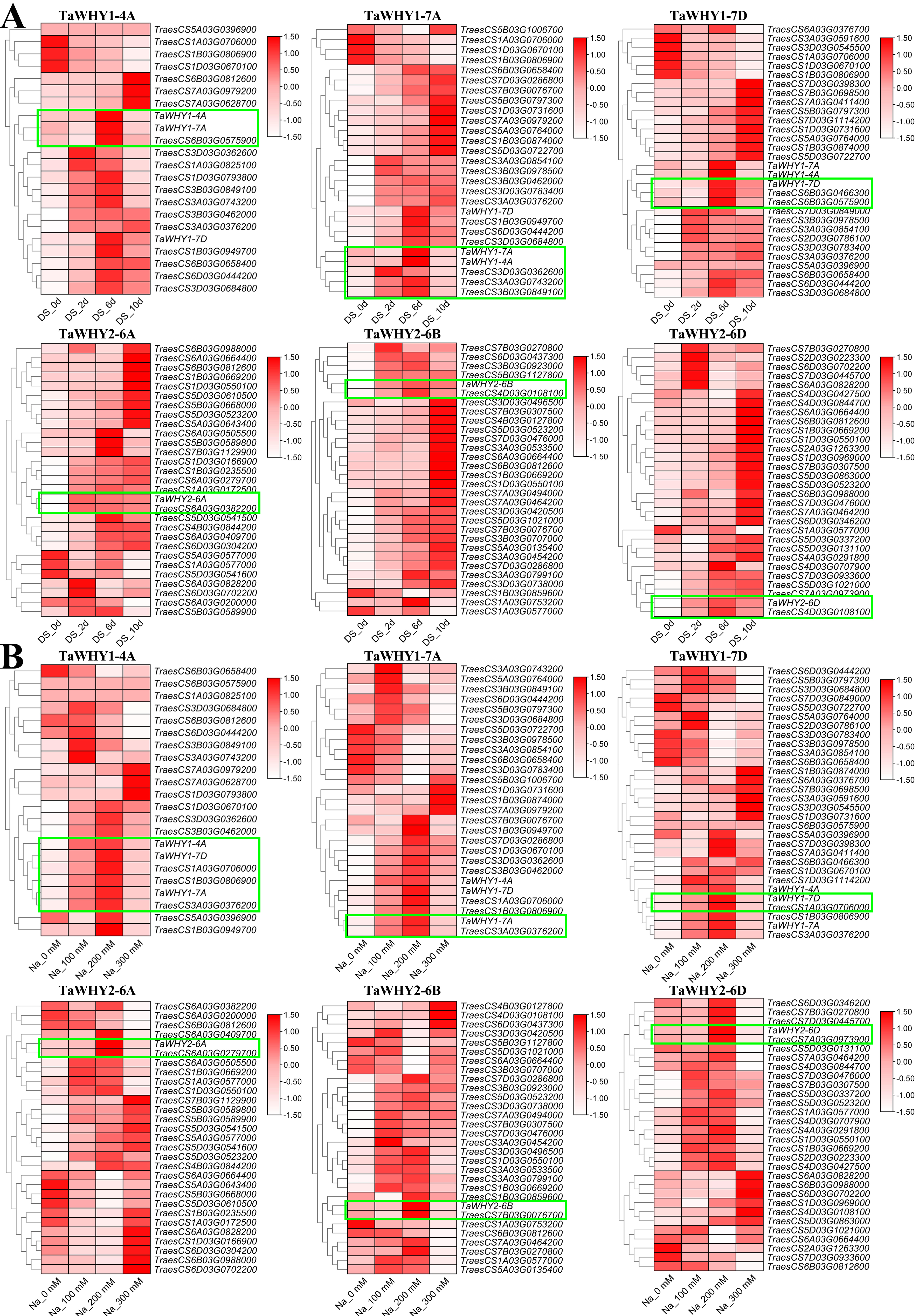

Supplement: Supplementary Figure 2 — The FPKM values of upstream transcription factors of TaWHYs under drought (A) and salt (B) stress. [file Image_2.jpeg]

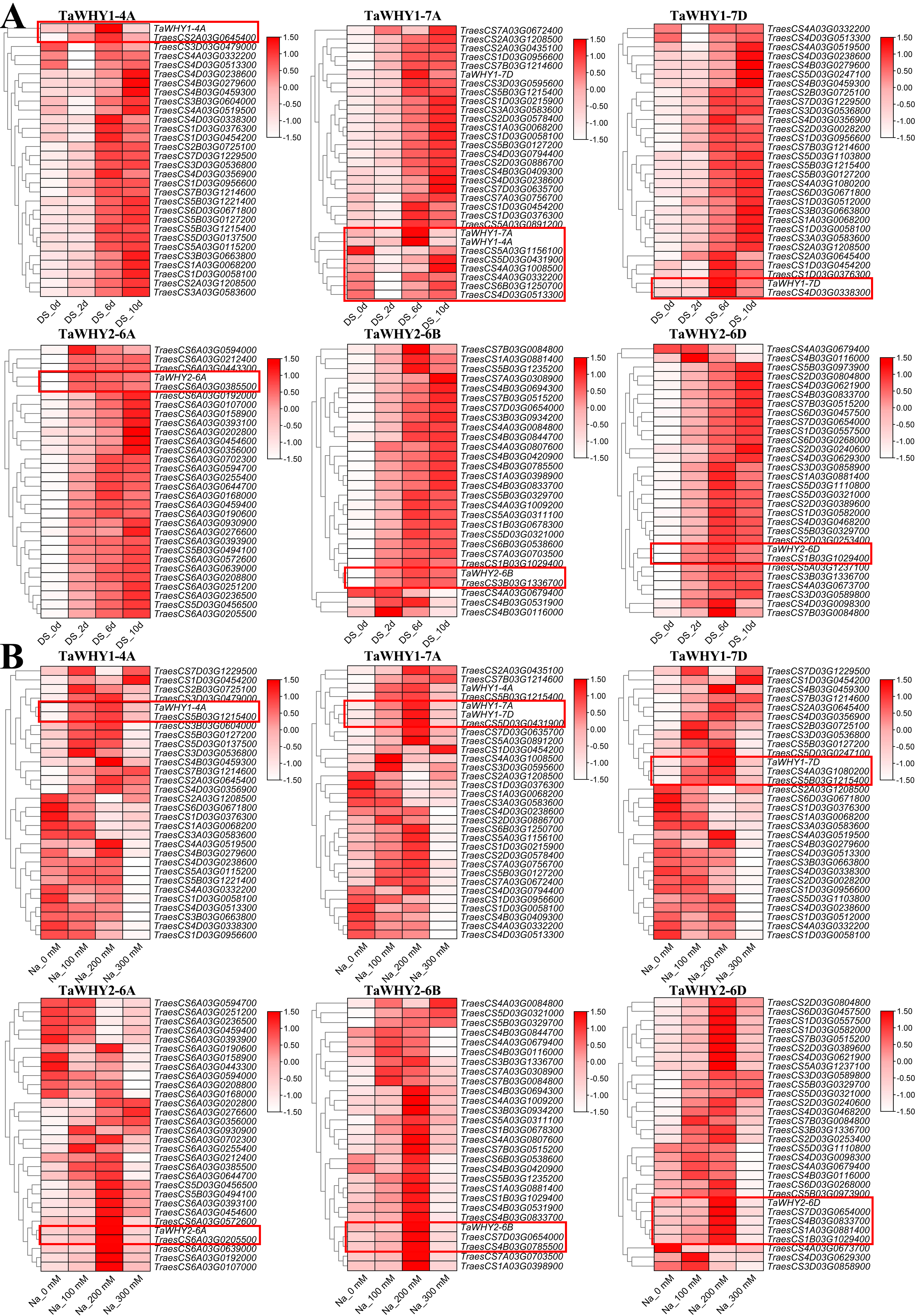

Supplement: Supplementary Figure 3 — The FPKM values of downstream target genes of TaWHYs under drought (A) and salt (B) stress. [file Image_3.jpeg]

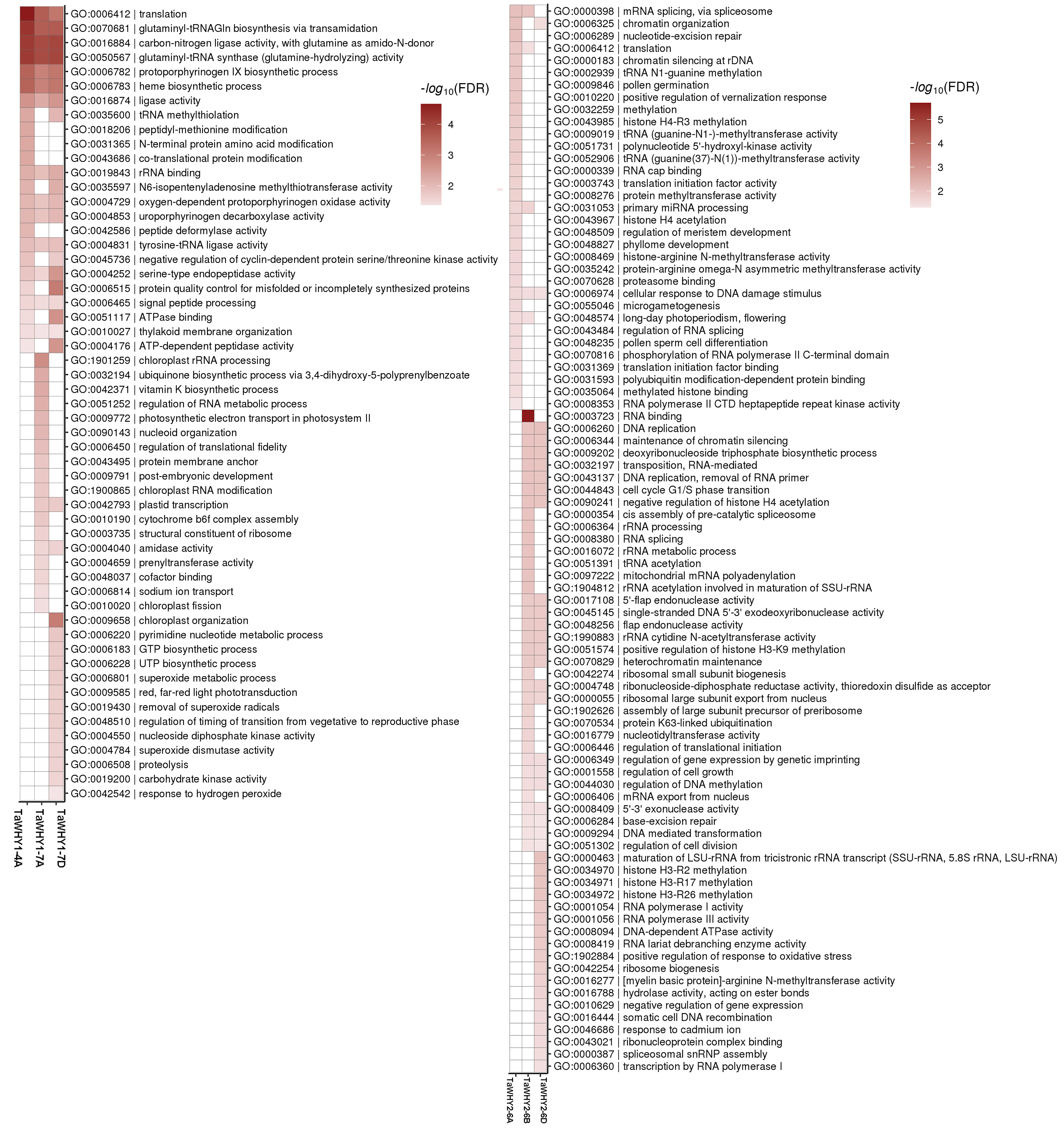

Supplement: Supplementary Figure 4 — GO enrichment analysis on the downstream target genes of TaWHY genes. [file Image_4.jpeg]

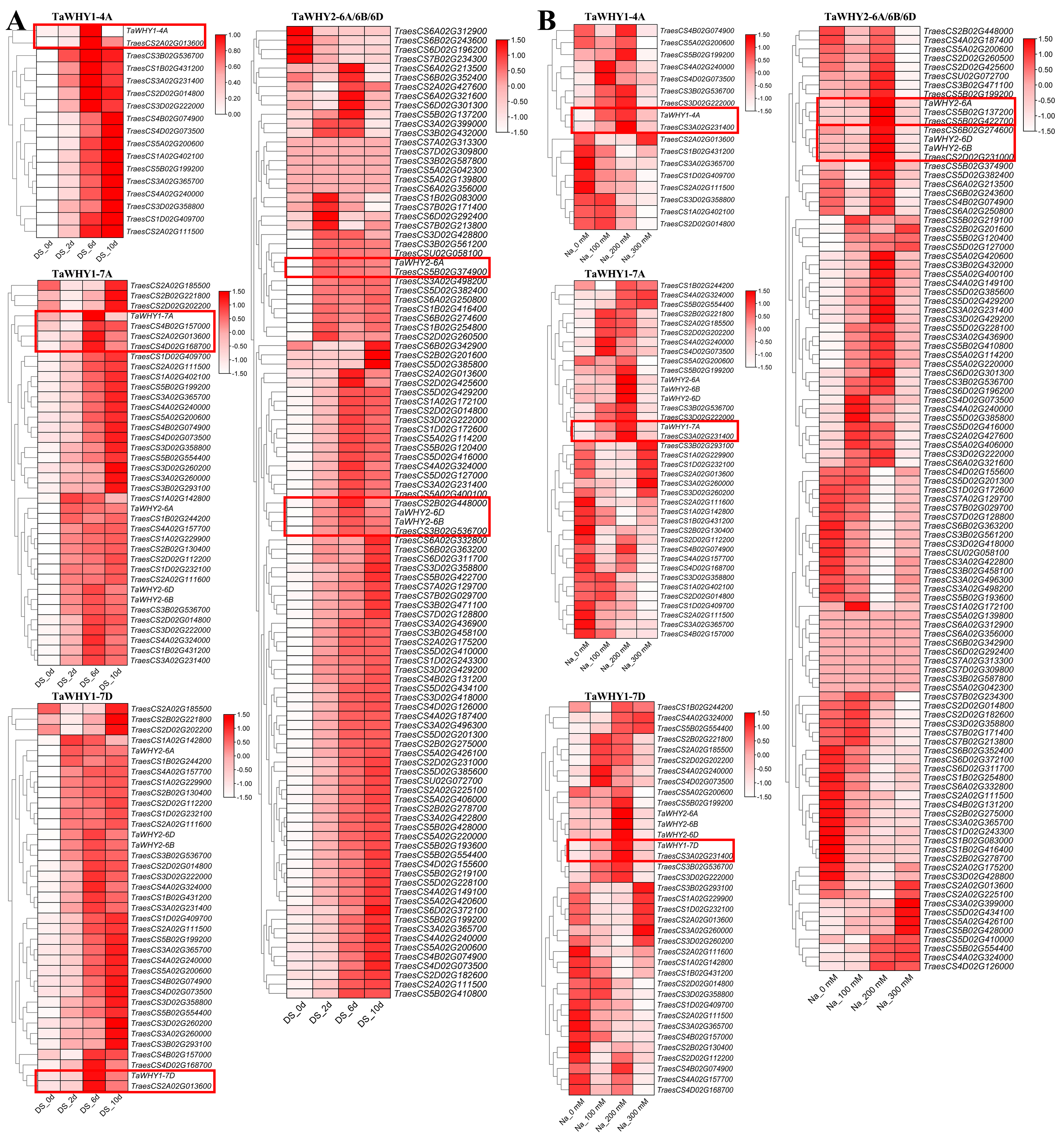

Supplement: Supplementary Figure 5 — The FPKM values of interacting protein of TaWHYs under drought (A) and salt (B) stress. [file Image_5.jpeg]

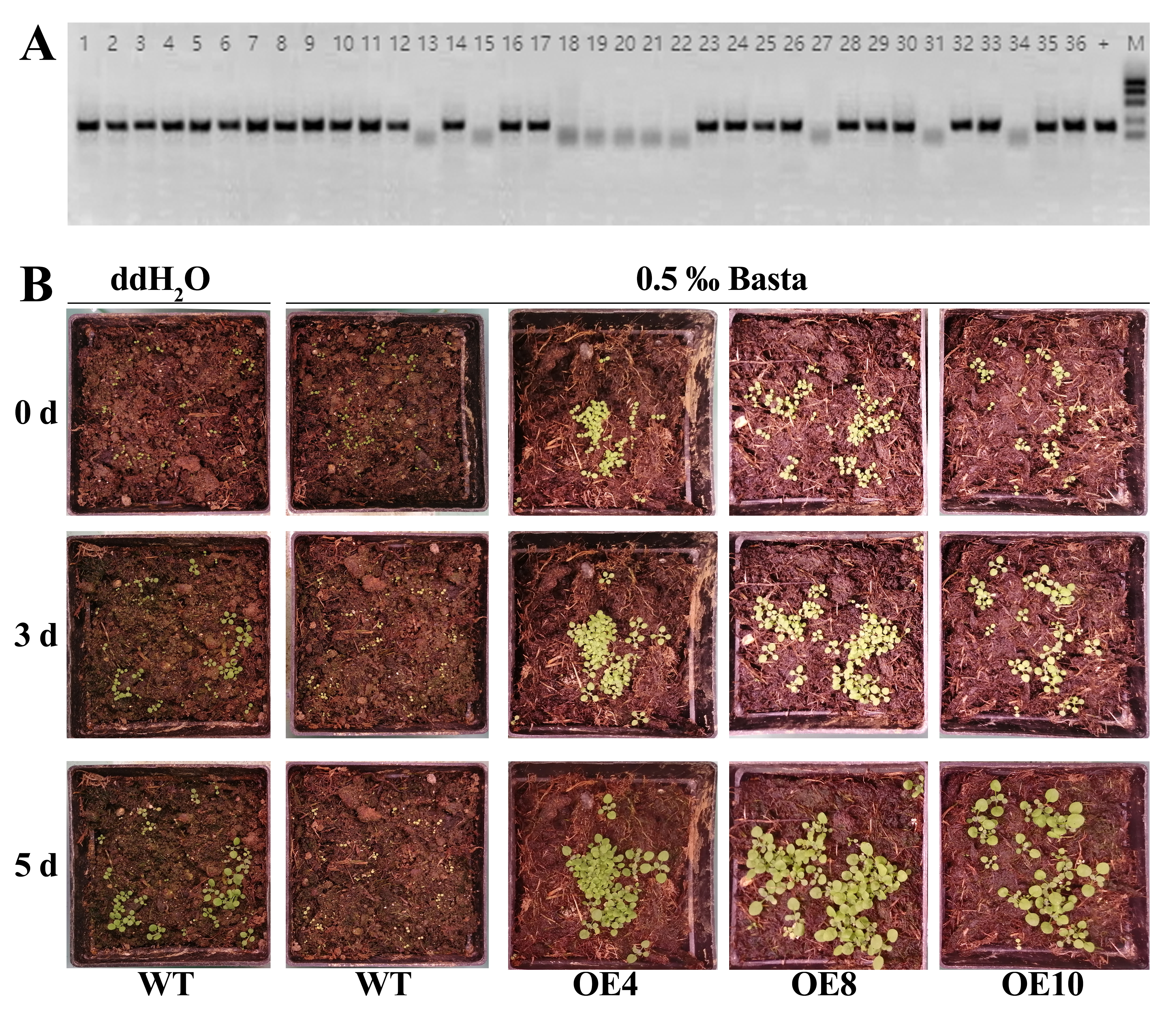

Supplement: Supplementary Figure 6 — The PCR detection (A) and screening (B) of 35S: TaWHY1-7D transgenic Arabidopsis. [file Image_6.jpeg]
